# Supplementary material for: Hyperthermic Intraperitoneal Chemotherapy for Primary or Recurrent Adrenocortical Carcinoma. A Single Center Study
Source: Cancers (Basel). 2020 Apr 14;12(4):969. doi: 10.3390/cancers12040969 (PMC7226100; doi:10.3390/cancers12040969)
Supplement: Supplementary file 1 [file cancers-12-00969-s001.pdf]

Supplementary Materials

Hyperthermic Intraperitoneal Chemotherapy for Primary or Recurrent Adrenocortical Carcinoma. a Single Center Study

Guido Alberto Massimo Tiberio, Vittorio Ferrari, Zeno Ballarini, Giovanni Casole, Marta Laganà, Michele Gritti, Elisa Arici, Salvatore Grisanti, Riccardo Nascimbeni, Sandra Sigala, Alfredo Berruti and Arianna Coniglio

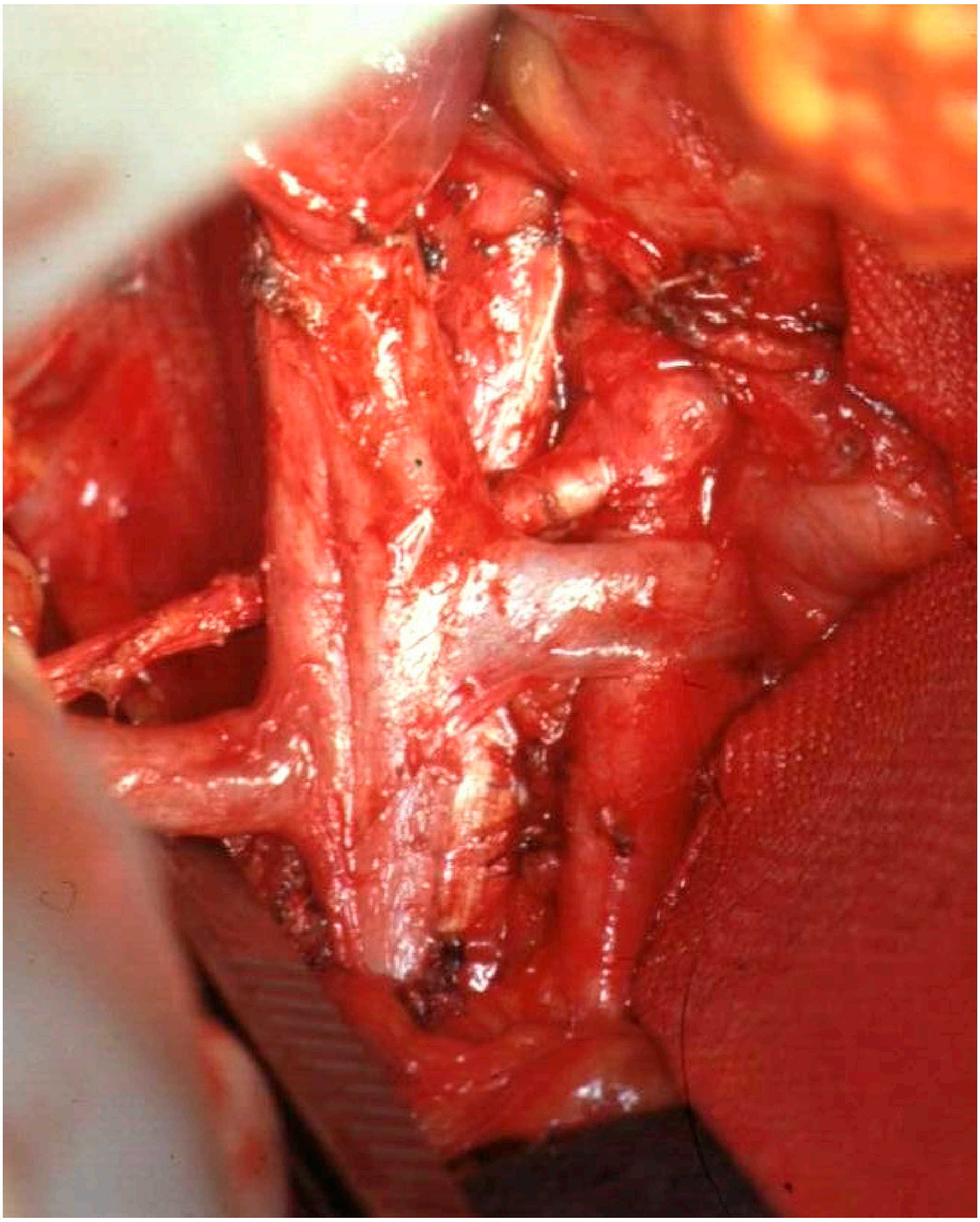

Figure S1. Panoramic view of surgical field after right regional adrenalectomy.

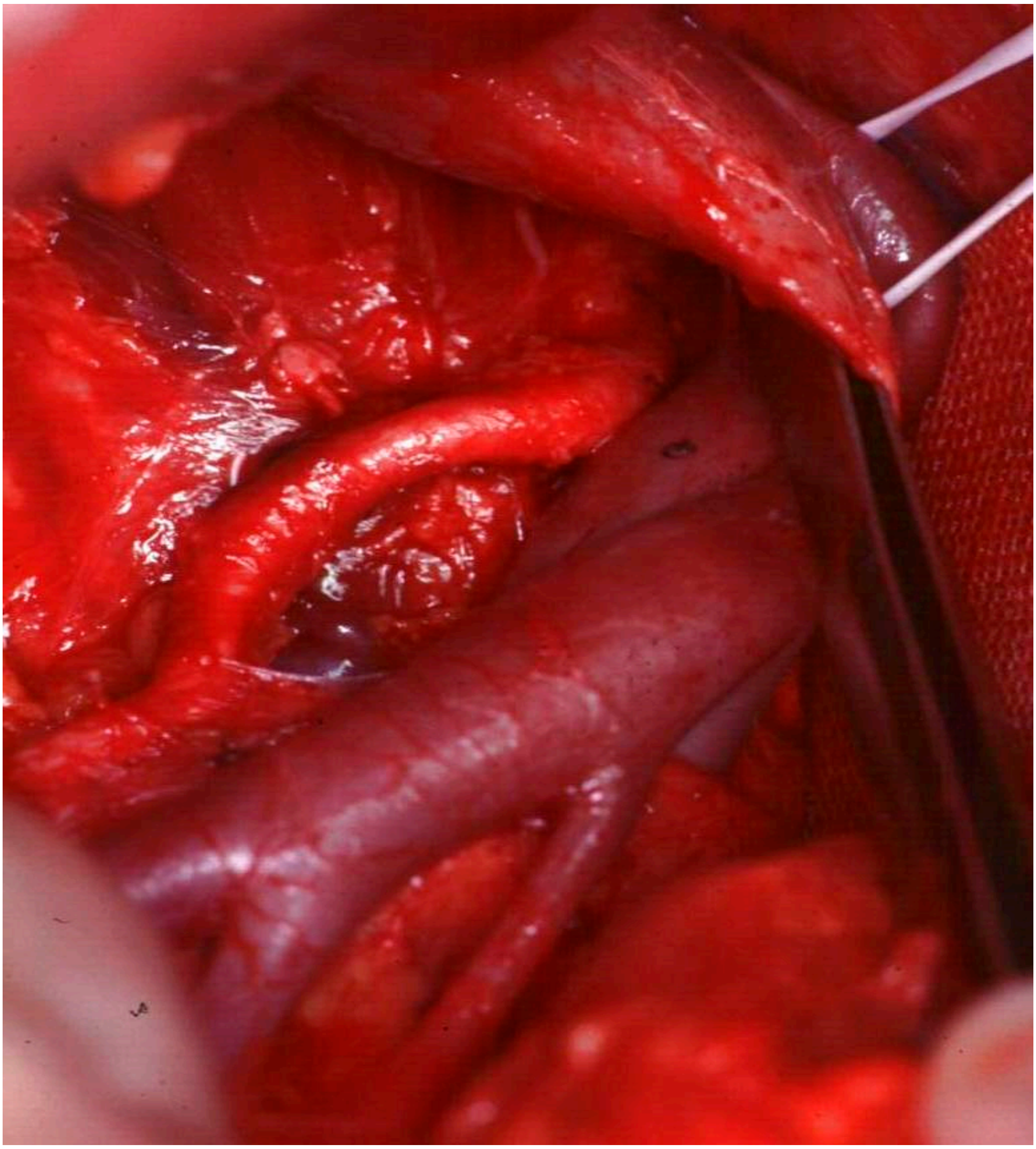

Figure S2. Same patient, particular. Renal vessels after regional adrenalectomy.

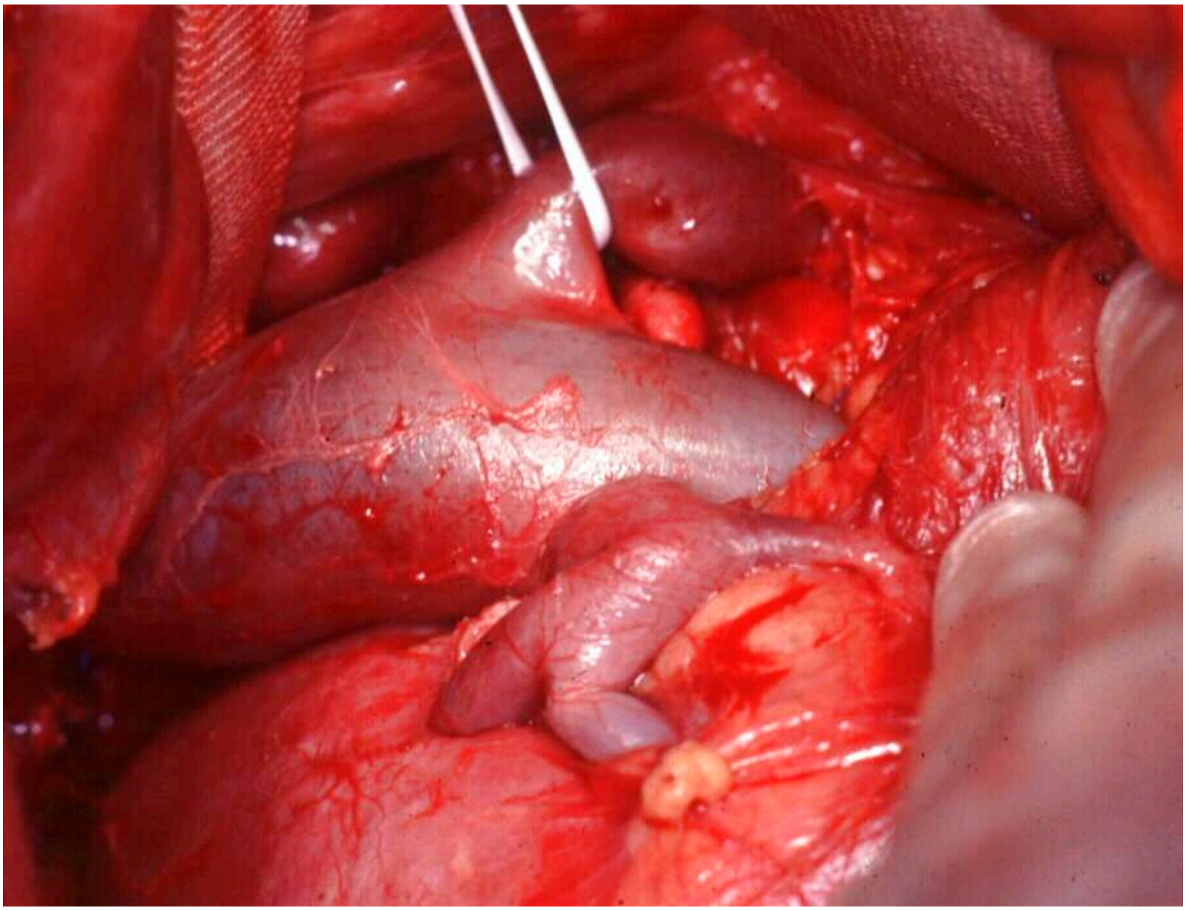

Figure S3. Same patient, particular: renal hilum after regional adrenalectomy.

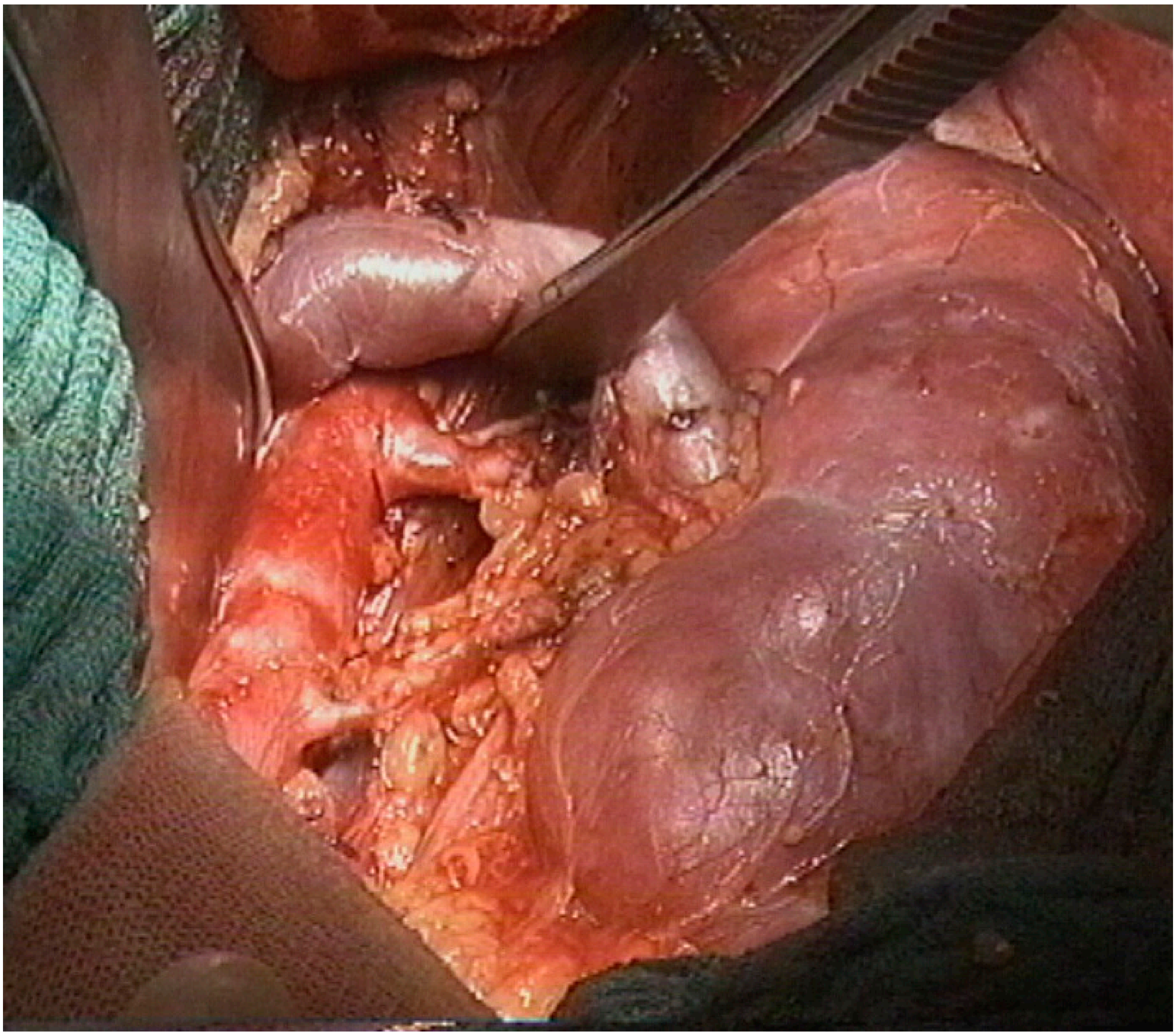

Figure S4. Panoramic view of surgical field after left regional adrenalectomy.

© 2020 by the authors. Licensee MDPI, Basel, Switzerland. This article is an open access article distributed under the terms and conditions of the Creative Commons Attribution (CC BY) license (http://creativecommons.org/licenses/by/4.0/).
